# The Differential Diagnosis of Intestinal Hæmorrhage

**Published:** 1912-11-23

**Authors:** 


### THE DIFFERENTIAL DIAGNOSIS OF INTESTINAL HÆMORRHAGE.

### II.—Some Symptoms of Obstruction and Disease.*

Hæmorrhage from the intestine occurs in from 3 to 6 per cent. of all cases of typhoid fever. There may be only a trace of blood in the motions or the bleeding may be so profuse as to prove fatal. The blood may be bright-red or dark, fluid or clotted, and if it has remained long in the intestine chocolate-coloured. It most frequently occurs from the end of the second week to the beginning of the fourth, and is usually coincident with the separation of the sloughs which leads to erosion of an artery. When it occurs earlier than the second week it is most probably the result of hyperæmia.

The ulcers are usually oval in shape and in the long axis of the intestine. They are most numerous in the lower part of the ileum immediately above the ileo-cæcal valve. The edges are thin and undermined, the muscular coat is exposed on the floor of the ulcer and in some cases the peritoneum.

### Three Kinds of Hæmorrhage.

Clinically, hæmorrhage from the intestine in typhoid fever may occur in three different ways:—

(*a*) The patient is lying in bed suffering from a disease which has been diagnosed as typhoid fever, and hæmorrhage occurs from the intestine as a complication. The onset of hæmorrhage may be indicated by restlessness, pallor, a sudden drop in the temperature, sometimes of as many as 6 or 8 degrees, a feeling of collapse, and an increase of pulse-rate with a fall in tension. When the bowels are next opened blood in large quantities may be passed.

(*b*) The patient is lying in bed suffering from a disease characterised by high temperature, rapid pulse and a general feeling of *malaise*, the diagnosis of typhoid fever not having been made. The occurrence of hæmorrhage from the intestine under these circumstances may be the first definite indication of the nature of the disease.

(*c*) Hæmorrhage from the bowel may be the first obvious sign of disease—the so-called ambulatory form of typhoid fever, but inquiry into the patient's medical history will probably elicit that for a period of two or three weeks he has been suffering from a general feeling of illness and slackness which commenced with headache and epistaxis, and that during this time he has had loss of appetite, a feeling of lassitude and weakness, and either diarrhœa or constipation. The temperature may be raised and the pulse may be dicrotic. The diagnosis may be confirmed by an examination of the blood, showing leucopenia and the blood serum giving a positive agglutination (Widal's) reaction.

### Embolism and Thrombosis of the Mesenteric Vessels.

Blood is usually passed per rectum in this condition. It may be dark in appearance, or the motions may be loose and contain bright-red blood and flakes of mucus. If the gut becomes gangrenous the stools are very fœtid. It occurs most frequently as a result of infective endocarditis or chronic valvular disease of the heart, especially mitral stenosis. Thrombosis may follow injury to the abdomen. There is a sudden attack of severe paroxysmal colicky pain in the abdomen, usually referred to the region of the umbilicus, which is probably due to a spasm of the intestines which follows the plugging of the vessel. Nausea and vomiting soon follow. The abdomen becomes tender, distended, and tympanitic. If the mesentery becomes much thickened from infiltration with blood a tumour may be felt and intussusception suspected. The patient soon becomes collapsed, the vomit becomes bile-stained, and even fæcal in character. The diagnosis from intestinal obstruction is difficult unless there is evidence of cardiac disease or of some other condition which is likely to have led to embolism.

### Malignant Disease of the Intestine.

Blood may be passed from the rectum in malignant disease of the intestine, but it is by no means a constant symptom. Cylindrical-celled carcinoma is the commonest form of growth; sarcoma is rare. The neoplasm usually encircles the gut, and the fibroid thickening which accompanies it causes stenosis, so that the symptoms are those of chronic intestinal obstruction. The growth, however, may be soft, and break down, involving the mucous membrane for several inches, and thus form a large ragged ulcer. It is in this form that hæmorrhage is most liable to occur, the motions being foul-smelling, dark, fluid, and containing blood clots and shreds of growth. An abdominal tumour may or may not be felt. The patient will be wasted, weak, and anæmic, and complain of abdominal pain. As the rectum and sigmoid flexure are the parts of the intestine most frequently affected by malignant disease, a digital and, if need be, a sigmoidoscopic, examination of the rectum and pelvic colon should always be made, and in this way the presence of the growth may be detected.

It should never be forgotten that malignant disease of the intestines or rectum may be secondary, not primary, and accordingly a careful search should always be made for any focus of growth in the breast, uterus, or elsewhere. Particularly in cases of carcinoma of the stomach, dissemination may occur and a growth may appear engrafted on the rectum and be palpable by rectal examination. So, too, a growth may extend into the intestine from a neighbouring region, for instance, the uterus; confusion from this source is hardly likely to arise, but it is well to keep the point in mind.

* The first article appeared on November 9.

